# Invasive grass (*Microstegium vimineum*) indirectly benefits spider community by subsidizing available prey

**DOI:** 10.1002/ece3.6752

**Published:** 2020-09-07

**Authors:** Andrew P. Landsman, Karin T. Burghardt, Jacob L. Bowman

**Affiliations:** ^1^ National Park Service United States Department of the Interior Hagerstown Maryland USA; ^2^ Department of Entomology University of Maryland College Park Maryland USA; ^3^ Department of Entomology and Wildlife Ecology University of Delaware Newark Delaware USA

**Keywords:** Araneae, indirect effects, invasive species, Japanese stiltgrass, *Microstegium vimineum*, multi‐trophic interactions, predator–prey interactions

## Abstract

1. Invasive plant species cause a suite of direct, negative ecological impacts, but subsequent, indirect effects are more complex and difficult to detect. Where identified, indirect effects to other taxa can be wide‐ranging and include ecological benefits in certain habitats or locations.
2. Here, we simultaneously examine the direct and indirect effects of a common, invasive grass species (*Microstegium vimineum*) on the invertebrate communities of understory deciduous forests in the eastern United States. To do this, we use two complementary analytic approaches to compare invaded and reference plots: (a) community composition analysis of understory arthropod taxa and (b) analysis of isotopic carbon and nitrogen ratios of a representative predatory spider species.
3. Invaded plots contained a significantly greater abundance of nearly all taxa, including predators, herbivores, and detritivores. Spider communities contained over seven times more individuals and exhibited greater species diversity and richness in invaded plots.
4. Surprisingly, however, the abundant invertebrate community is not nutritionally supported by the invasive plant, despite 100% ground cover of *M. vimineum*. Instead, spider isotopic carbon ratios showed that the invertebrate prey community found within invaded plots was deriving energy from the plant tissue of C_3_ plants and not the prevalent, aboveground *M. vimineum*.
5. *Synthesis and applications*. We demonstrate that invasive *M. vimineum* can create non‐nutritional ecological benefits for some invertebrate taxa, with potential impacts to the nutritional dynamics of invertebrate–vertebrate food webs. These positive impacts, however, may be restricted to habitats that experience high levels of ungulate herbivory or reduced vegetative structural complexity. Our results highlight the importance of fully understanding taxon‐ and habitat‐specific effects of invading plant species when prioritizing invasive species removal or management efforts.

## Graphical abstract

The indirect effects of invasive plant species are complex, difficult to detect, and in some cases may provide ecological benefits in certain habitats. We identified positive effects of an invasive grass species on the abundance, richness, and diversity of a forest spider community, mediated through available insect prey. Such ecological benefits are certainly habitat‐ and species‐specific, which highlights the importance of understanding indirect effects when managing invasive species.

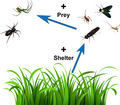

## 1 INTRODUCTION

Direct effects of one species on another can be relatively easy to quantify, particularly when considering simplified, two species interactions. However, such straightforward exchanges are rarely found in nature, with secondary and tertiary effects pervading into subsequent trophic levels and affecting multiple species (Preisser, Bolnick, & Benard, 2005; Werner & Peacor, 2003). Indirect interactions of one species on another through a third species can affect species' density and can impose behavioral changes to the indirectly affected species (Abrams, 1995; Werner & Peacor, 2003). Given the prevalence and strength of indirect species interactions, these relationships can often exert more influence over ecological communities that can direct interactions between a predator and herbivore (Seibold, Cadotte, MacIvor, Thorn, & Müller, 2018) and have been documented in various aquatic and terrestrial habitats (Fletcher et al., 2019; Vilá et al., 2011). Despite their ecological significance, indirect effects among species can be complex and difficult to detect, and as a result are often understudied and overlooked.

The ecological consequences of the invasion of certain non‐native plant species are well‐supported throughout the literature, with direct, negative effects documented on a wide range of native taxa. These impacts occur when invasive plants directly reduce available habitat and survival of native plant species (Vilá et al., 2011) or directly affect availability of plant material used by birds and invertebrates for food, shelter, and oviposition (Ballard, Hough‐Goldstein, & Tallamy, 2013; Bultman & DeWitt, 2008; Burghardt & Tallamy, 2013; Heleno, Ceia, Ramos, & Memmott, 2009; Meyer, Schmidt, & Robertson, 2015; Mollot, Pantel, & Romanuk, 2017). Beyond directly impacting both the behavior and density of other taxa, invasive plant species can also cause negative indirect effects mediated through alteration of the behavior and density of other, often herbivorous, species (Vilá et al., 2011; White, Wilson, & Clarke, 2006). Nevertheless, there exists an expanding literature detailing both direct and indirect impacts considered positive or beneficial for a species or ecological community resulting from non‐native plant invasion (McCary, Mores, Farfan, & Wise, 2016; Tymkiw, Bowman, & Shriver, 2013). Some invading plant species, often with large and showy inflorescences, can increase floral density and food availability for pollinators throughout the season and potentially at times of reduced floral availability (Davis, Kelly, Maggs, & Stout, 2018; Russo, Nichol, & Shea, 2016). The complex and novel architecture of many invasive plant species can also provide enhanced structure for primary consumers to effectively hide from predators (Dutra, Barnett, Reinhardt, Marquis, & Orrock, 2011; Malo et al., 2012). Contrastingly, changes in plant structure can also directly benefit actively hunting predators (Loomis, Cameron, & Uetz, 2014) or those that use passive hunting techniques (Dudek, Michlewicz, Dudek, & Tryjanowski, 2016; Pearson, 2009). Several examples exist whereby predator populations are augmented due to the change in plant structure caused by the invading plant species, ultimately causing indirect suppression of plant consumer populations. These aforementioned studies examined impacts of common invasive plant species of the United States (U.S.): *Centaurea maculosa* (spotted knapweed) in the West, *Microstegium vimineum* (Japanese stiltgrass) in the South, and *Alliaria petiolata* (garlic mustard) in the Northeast, respectively. However, these examples focused on pairs of morphologically similar predator species (DeVore & Maerz, 2014; Pearson, 2009, 2010) or on specific predatory functional guilds (Smith‐Ramesh, 2017) and not on predatory communities of the forest understory as a whole.

*Microstegium vimineum* is a grass species from eastern Asia that invades forest edges and disturbed habitats in the eastern half of the U.S. (Flory, Long, & Clay, 2011; Huebner, 2010). The species forms dense mats across the forest floor by spreading from stolons, ultimately reducing native tree seedling density, growth, and diversity (Oswalt, Oswalt, & Clatterbuck, 2007; Brewer, 2011), as well as overall native plant species cover (Adams & Engelhardt, 2009). The forest floor in invaded habitats in Maryland, often in areas with high densities of vertebrate herbivores, is generally simplified in structure, due in part to the growth habit of *M. vimineum* (Civitello, Flory, & Clay, 2008; Landsman pers. obs.). Given its growth habit and stand density, the presence of *M. vimineum* also alters abiotic characteristics of the near‐ground forest environment. The forest floor experiences an increase in solar irradiation within *M. vimineum* stands, which decreases relative humidity and increases microhabitat temperatures (Civitello et al., 2008). Such climatic changes subsequently alter the ability of *M. vimineum* patches to host invertebrate populations: *M. vimineum* ground cover has been shown to reduce the overall diversity of soil microarthropods by greatly increasing the abundance of mites and subsequently reducing community evenness (McGrath & Binkley, 2009). The abundance of cicadellidae planthoppers, as well as acridid and gryllid Orthoptera, was also found to be higher in *M. vimineum* patches (Marshall & Buckley, 2009). Conversely, *M. vimineum* can also reduce the abundance of Blattodea and chrysomelid beetles, as well as the abundance and survival of hard tick species in the Ixodidae (Civitello et al., 2008; Marshall & Buckley, 2009).

*Microstegium vimineum* directly affects forest floor invertebrates by physically altering their habitat; however, knowledge of subsequent indirect effects to the predators that utilize those affected invertebrates resulting from invasion‐driven forest floor changes is lacking. Physiognomic changes in structural complexity resulting from invasive plant species with similar, mat‐forming growth habits have been shown to alter community structure, composition, and species abundance of the spider community (Bultman & DeWitt, 2008; Wolkovich, Bolger, & Holway, 2009). Given the importance of forest spiders as an intermediate link between vertebrate and invertebrate food webs, the indirect impacts of invading *M. vimineum* on forest‐dwelling spider communities have the potential to augment or depress prey densities, affect vertebrate predator populations, and alter the nutritional dynamics of invertebrate and vertebrate food webs (Gunnarsson, 1996, 2007; Miyashita & Takada, 2007; Philpott, Greenberg, Bichier, & Perfecto, 2004; Spiller & Schoener, 1988; Walters, Mills, Fritz, & Raikow, 2010).

Here, our objective was to better understand the direct and indirect impacts that the invasive *M. vimineum* has on the invertebrate prey and predator communities and to demonstrate whether such invasions have the potential to affect predator–prey interactions in the forest understory. As *M. vimineum* invades, it can alter near‐ground vertical plant structure and the availability of palatable food resources, both of which may directly and indirectly alter the composition of the invertebrate community. We hypothesized that the abundance of herbivorous insects would decrease in *M. vimineum* patches due to suppression of native vegetation and food resources, and that, conversely, dipteran species in detrital food webs would prefer the sheltered microhabitat created by dense *M. vimineum* stands. We similarly predicted changes to the spider community in invaded habitats: While spider richness and diversity would not change within *M. vimineum*, we expected to see changes in community composition as alterations to near‐ground plant structure negatively impact web‐building taxa and benefit active hunting spiders. Finally, we hypothesized that web spider isotopic nitrogen signatures would reflect the greater proportion of available detritivorous insects in spider diet and that spider isotopic carbon would reflect the greater relative contribution of C_4_ *M. vimineum* biomass in invaded areas.

## 2 MATERIALS AND METHODS

Our study area included deciduous forests of U.S. National Park Service lands located in Washington, Frederick, and Montgomery Counties, Maryland: Antietam National Battlefield (Antietam), Monocacy National Battlefield (Monocacy), and portions of the Chesapeake and Ohio Canal National Historical Park (Great Falls). Forests were similar in that they contained multiple invasive plant species and scant understory vegetation, in part due to dense white‐tailed deer (*Odocoileus virginianus*) populations (46–66 deer/km^2^) (U.S. National Park Service, unpubl. data). Dominant trees included native *Acer* spp., *Carya* spp., *Fagus grandifolia*, and *Liriodendron tulipifera* while the understory consisted of mostly *Lindera benzoin*, *Asimina triloba*, and exotic plants such as *M. vimineum*, *Alliaria petiolata*, and *Rosa multiflora*.

To study invertebrate community response to *M. vimineum*, we used a paired plot design across our study area. Within each of the three parks, we opportunistically located 16 patches of *M. vimineum* at least 10 m by 10 m in size, and at least 10 m away from the forest edge. Sites were selected that contained greater than or equal to 80% visual ground cover of *M. vimineum*. We established the center of a 6 m by 6 m square plot at the approximate center of the patch. Paired with each *M. vimineum* plot, we also established a reference plot of the same size, without *M. vimineum*. We used random integers to select an azimuth from which to establish reference plots, 20 m from the edge of the *M. vimineum* patch, at least 10 m from the forest edge, and within the same vegetation community type. Within individual square plots for both invaded and reference habitats, we estimated ground cover of *M. vimineum* in four 1 m^2^ square subplots, 1 m away from the plot center in the four cardinal directions, and used the mean ground cover estimate for each plot. We also measured understory vegetative structure using a 2.0 m tall profile board placed in the center of the plot. We estimated the percentage of the board that was obscured by vegetation between 0.5 and 2.0 m in height and used the mean of the four values for each plot in analyses.

We conducted vacuum sampling within each individual square plot in mid‐July 2017. We vacuumed insects and arachnids between 0.5 and 2.0 m above the ground surface in order to avoid forest floor and fossorial taxa. We used a commercially available leaf blower and vacuum (Black+Decker LSWV36) with a 2‐gallon paint strainer bag affixed to the intake tube to vacuum vegetative surfaces, spider webs, and other spaces between vegetation in each plot. We vacuumed throughout the entirety of each 36 m^2^ plot for a standardized 7 min. After sampling, we euthanized collected arthropods using ethyl acetate, removed vegetative debris, and placed invertebrates in 70% ethyl alcohol. We identified insects to order using Triplehorn and Johnson (2005). However, certain orders were further subdivided if palatability to forest spiders greatly differed within groups: Weevils (Curculionidae) were classified separately from other Coleoptera, predatory assassin bugs (Reduviidae) and damsel bugs (Nabidae) were separated from herbivorous Hemiptera, ants (Formicidae) were considered distinct from the other Hymenoptera, and Lepidoptera were subdivided into caterpillars and adults. Spiders were identified to genus or species when possible using Ubick, Paquin, Cushing, and Roth (2017). Any specimens not identified to this taxonomic resolution, including those too damaged for identification or recently hatched individuals, were excluded from community analysis and analyses of diversity and richness.

For a closer examination of changes in nutritional dynamics, we also specifically sampled a representative orb‐weaving spider common to eastern deciduous forests (Tetragnathidae: *Leucauge venusta* Walckenaer 1841) prior to vacuum sampling for the remaining invertebrate community. We sampled *L. venusta* as this species was commonly found throughout the study area and has a wide geographic distribution across much of eastern North America. This species spins a relatively horizontal orb web with attachment points in low‐growing vegetation in wooded areas. *L. venusta* and other relatively small orb‐weaving spiders prey mostly on flies (Diptera), leafhoppers (Hemiptera: Cicadellidae), and other small, alate true bugs, and beetles (Coleoptera). We collected mature female spiders by hand from plots in early July 2017. Samples were immediately frozen and individually dried for 24 hr at 60°C. We then weighed the dry spider samples to obtain body mass. Spiders were then individually ground, homogenized, and encapsulated. We analyzed individual spiders for δ^15^N and δ^13^C using a continuous flow isotope ratio mass spectrometer (DELTA V Plus; Thermo Fisher Scientific) and elemental analyzer (NC 2,500, Carlo Erba; 95% CI ±0.5‰). Isotopic nitrogen ratios can provide information on a predator's diet, while isotopic carbon can reveal photosynthetic pathways in sampled organisms: *M. vimineum*, a C_4_ species, maintains δ^13^C levels between −13‰ and −15‰, while C_3_ plants generally have δ^13^C values near −27‰ (Bradford et al., 2010; Hyodo, 2015). Analyses were conducted at the Central Appalachians Stable Isotope Facility at the University of Maryland Center for Environmental Science's Appalachian Laboratory. Isotopic carbon results were expressed in parts per mille relative to Vienna PeeDee Belemnite, with isotopic nitrogen reported in parts per mille relative to atmospheric nitrogen.

Data manipulation and statistical analyses were performed using R 3.4.4 (R Core & Team, 2018). We used linear regression to analyze relationships between spider δ^15^N and δ^13^C and park unit, the presence of *M. vimineum*, and their interaction. For each response variable, we first included understory plant structure as an additional covariate in the model. Likelihood ratio tests showed that plant structure was never an important explanatory covariate. Thus, we excluded this metric from all analyses. Similarly, we also regressed the Shannon–Weiner diversity, abundance, and richness of spider and insect communities in models with the same independent variables. For those linear models and variables that exhibited non‐normality, including models with spider body mass and isotopic carbon and nitrogen, we employed a square root transformation of the dependent variable. For count data, including invertebrate abundance and richness, we used generalized linear models with a negative binomial probability distribution using *glm.nb* in the *MASS* package (Venables & Ripley, 2002). We obtained *p*‐values from the likelihood ratio test statistic using *anova.glm*. For spider and insect community data, we calculated the Euclidean distance between taxa within community matrices after Hellinger transformation (Borcard, Gillet, & Legendre, 2011; Legendre & Legendre, 2012; Rao, 1995). We used the Euclidean distance of Hellinger‐transformed data as these data are metric and considered robust in ordination analyses (Legendre & Gallagher, 2001). We then conducted permutational multivariate analysis of variance using *adonis2* in the *vegan* package to understand how communities differ in patches of *M. vimineum* after testing for multivariate homogeneity of variance using *betadisper* and *anova.betadisper* (Anderson, Ellingsen, & McArdle, 2006; McArdle & Anderson, 2001; Oksanen et al., 2017). To test for significance, we examined the marginal effects of variables after 20,000 permutations.

## 3 RESULTS

**FIGURE 1:**
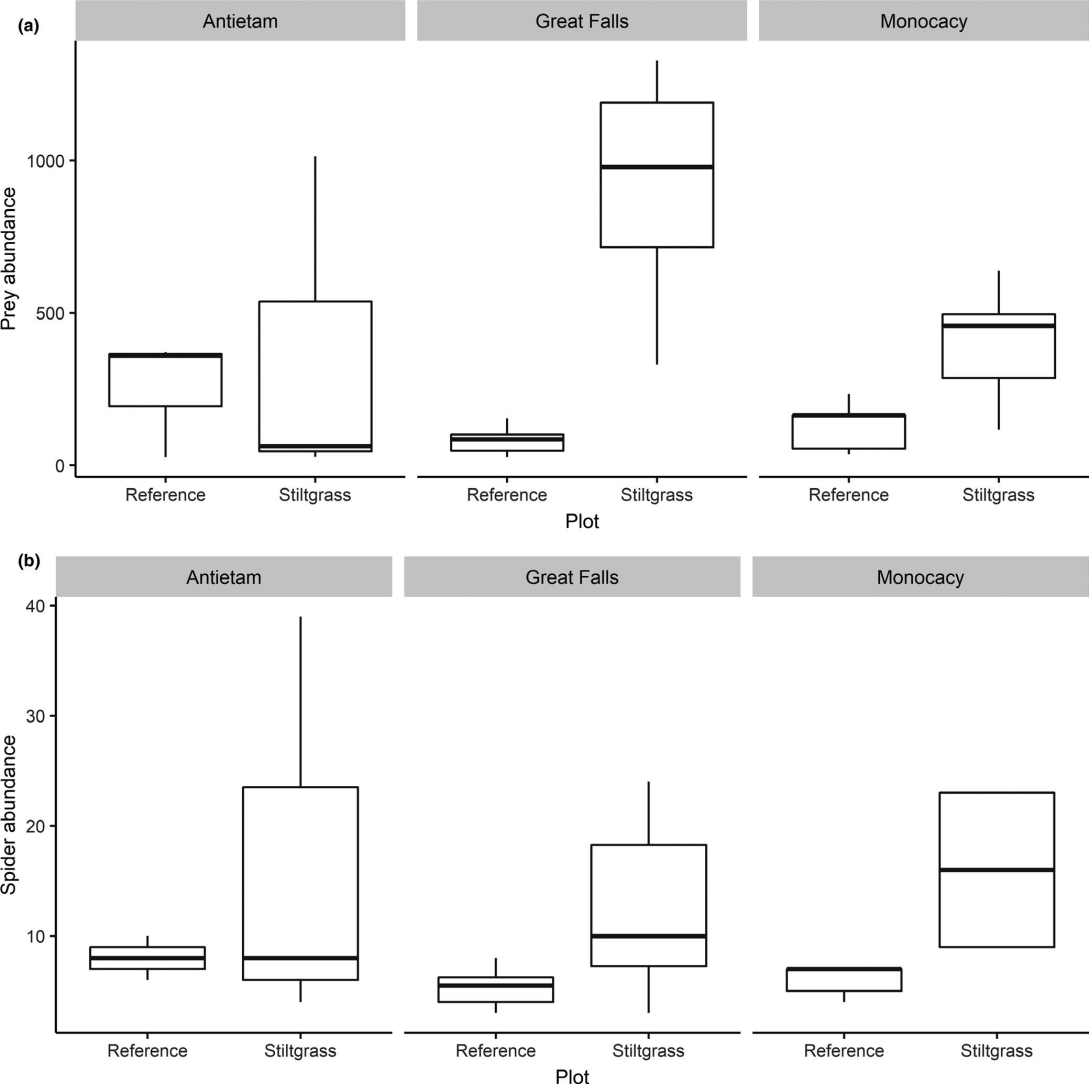
Boxplots showing effects of *Microstegium vimineum* (Japanese stiltgrass) on the abundance of (a) insect prey and (b) adult and subadult spiders, replicated in three parks

Ground cover of *M. vimineum* was 100% in all invaded subplots. Reference plots did not contain *M. vimineum* and were sparsely vegetated except for a single reference plot at Antietam, where dense vegetation consisted of non‐native shrubs (*Berberis thunbergii*) and early successional plant species (*Asimina triloba* and *Verbesina alternifolia*). Understory vertical vegetative structure was minimal in both invaded and reference plots, with mean plant cover of 2.25% between 0.5 and 2.0 m; however, the aforementioned reference plot at Antietam exhibited 35.63% vertical vegetative cover. *M. vimineum* plots showed mean vertical vegetative cover of 0.27% while reference plots contained mean 4.23% cover. Across all plots, we collected a total of 15,453 invertebrates in 19 orders, including three arachnid groups: mites (Acari), spiders (Araneae), and harvestmen (Opiliones). The total number of invertebrates, including adult and juvenile spiders, was greater in plots within *M. vimineum* patches (*G^2^* = 44.007; *p* < .0001), with 12,879 individuals collected as compared to 2,574 collected in corresponding reference plots (Table 1). Invaded and reference plots at Antietam contained mean ± *SE* 360.5 ± 173.0 invertebrates and plots at Monocacy contained 360.9 ± 87.7 invertebrates, whereas Great Falls included 604.9 ± 153.5 individuals. The abundance of prey palatable to forest spiders, including beetles, springtails, flies, and herbivorous true bugs, was similarly greater in invaded patches (*G^2^* = 35.883; *p* < .0001; Figure 1a). Most taxa, when assessed individually, also exhibited significant positive relationships with the presence of *M. vimineum*. This was often paired with a significant interaction term between *M. vimineum* and individual park (Table 2). Flies were more abundant in *M. vimineum*, with a total of 9,354 flies collected in invaded plots while 1,682 were found in reference plots (*G^2^* = 33.034; *p* < .0001). Herbivorous true bugs displayed a similar relationship, with 804 and 287 individuals collected in invaded and reference plots, respectively (*G^2^* = 25.188; *p* < .0001). Beetles, excluding the weevils, were not correlated with the presence of *M. vimineum* (*G^2^* = 0.335; *p* = .5626). We also found significant differences in community composition between *M. vimineum* plots and their paired reference plots (*F*_1,26_ = 5.970; *p* = .00005) and among parks (*F*_2,26_ = 3.069; *p* = .0004).

**TABLE 1:** Mean abundance of invertebrate groups within invaded and reference plots throughout study area

Taxon	Mean abundance ± *SE* in invaded plots	Mean abundance ± *SE* in reference plots
Araneae (total)	84.63 ± 18.50	11.63 ± 1.21
Araneae (adults/subadults)	14.25 ± 2.41	6.06 ± 0.46
Araneae (juveniles)	70.38 ± 17.95	5.56 ± 1.05
Araneae (orb web)	10.44 ± 2.03	5.13 ± 0.49
Araneae (space web)	2.88 ± 0.53	0.81 ± 0.29
Araneae (hunting)	1.00 ± 0.30	0.19 ± 0.10
Acari	0.31 ± 0.12	0.06 ± 0.06
Coleoptera	4.44 ± 0.80	5.38 ± 0.98
Collembola	7.13 ± 2.58	0.25 ± 0.11
Diptera	584.63 ± 106.99	105.13 ± 23.16
Hemiptera	50.25 ± 9.77	17.94 ± 4.81
Hemiptera (predatory)	1.06 ± 0.36	0.38 ± 0.16
Hymenoptera	10.31 ± 1.54	6.88 ± 1.44
Hymenoptera (ants)	34.63 ± 14.29	2.81 ± 0.53
Lepidoptera	4.06 ± 0.69	1.31 ± 0.33
Opiliones	4.94 ± 1.33	1.50 ± 0.52
Orthoptera	6.94 ± 1.20	0.63 ± 0.32
Psocoptera	3.81 ± 1.08	5.31 ± 1.50
Thysanoptera	1.63 ± 0.63	0.81 ± 0.51
Total invertebrates	804.94 ± 132.00	160.88 ± 30.57

**TABLE 2:** Significance of sampling location (Park), presence of *Microstegium vimineum*, and their interaction on total abundance of insect and arachnid taxa

Taxon	Park		Presence of *M. vimineum*		Park*M. vimineum	
	*G^2^*	*p*	*G^2^*	*p*	*G^2^*	*p*
Araneae (total)	1.759	0.4150	66.504	**<.0001**	6.748	**.0343**
Araneae (adults/subadults)	2.351	0.3087	20.553	**<.0001**	0.250	.8827
Araneae (juveniles)	2.610	0.2712	54.703	**<.0001**	8.179	**.0167**
Araneae (orb web)	1.546	0.4615	11.013	**.0009**	1.386	.5001
Araneae (space web)	4.022	0.1338	18.847	**<.0001**	11.282	**.0036**
Araneae (hunting)	2.180	0.3362	9.765	**.0018**	7.014	**.0300**
Acari	2.560	0.2781	2.911	.0880	2.634	.2679
Coleoptera	1.904	0.3861	0.335	.5626	2.292	.3179
Collembola	7.171	**0.0277**	29.412	**<.0001**	6.473	**.0393**
Diptera	2.672	0.2629	33.034	**<.0001**	9.686	**.0079**
Hemiptera	16.309	**0.0003**	25.188	**<.0001**	4.371	.1124
Hemiptera (predatory)	1.996	0.3687	5.482	**.0192**	4.380	.1119
Hymenoptera	1.877	0.3913	3.853	**.0497**	2.048	.3592
Hymenoptera (ants)	4.307	0.1161	27.543	**<.0001**	10.390	**.0055**
Lepidoptera	14.166	**0.0008**	23.614	**<.0001**	8.959	**.0113**
Opiliones	11.324	**0.0035**	10.904	**.0010**	0.073	.9642
Orthoptera	5.166	0.0756	51.015	**<.0001**	9.975	**.0068**
Psocoptera	2.283	0.3194	0.293	.5883	5.096	.0782
Thysanoptera	5.616	0.0603	4.968	**.0258**	17.366	**.0002**
Total invertebrates	2.670	0.2632	44.007	**<.0001**	10.372	**.0056**

Note

Bold text indicates significance at *α* = 0.05.

**FIGURE 2:**
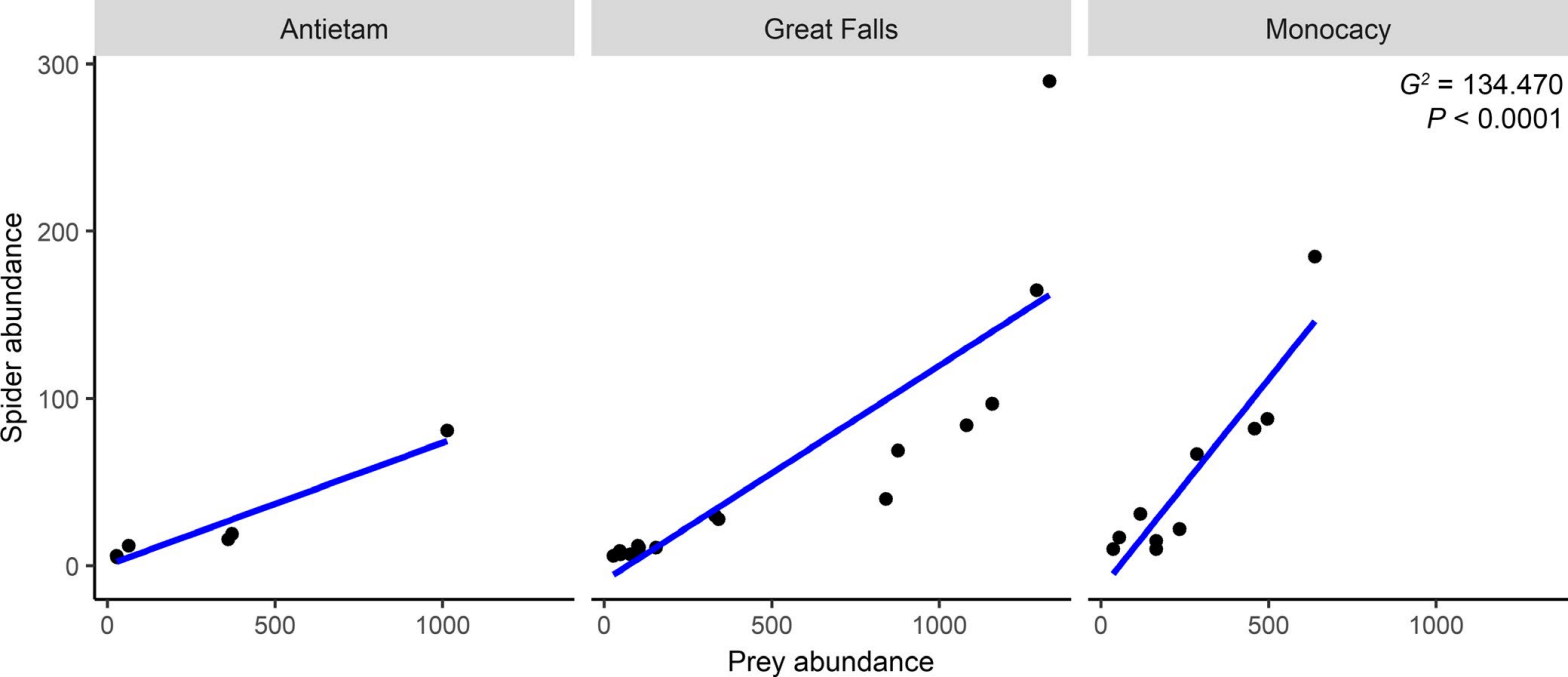
Relationship between insect prey and total spider abundance within plots at the three sampled parks

Spider species diversity increased within *M. vimineum* plots (*F*_1,26_ = 13.736; *p* = .0010) and was positively correlated with prey abundance (*F*_1,26_ = 4.563; *p* = .0422). Similarly, species richness was greater both within *M. vimineum* (*F*_1,26_ = 15.788; *p* = .0005) and with more prey (*G^2^* = 9.881; *p* = .0017). *M. vimineum* patches contained more orb web‐building (*G^2^* = 11.013; *p* = .0009), space web‐building (*G^2^* = 18.847; *p* < .0001), and hunting (*G^2^* = 9.765; *p* = .0018) spiders, as well as greater total spider abundance both with juveniles included (*G^2^* = 66.504; *p* < .0001) and without juvenile spiders (*G^2^* = 20.553; *p* < .0001; Figure 1b). We collected 325 adult and subadult spiders compared to 1,215 spiderlings and unidentified juveniles. Spider abundance increased with elevated prey densities (*G^2^* = 30.707; *p* < .0001). Total spider abundance, when including juveniles, exhibited even stronger positive correlations (*G^2^* = 134.470; *p* < .0001; Figure 2). Spider diversity, richness, and abundance did not differ among parks. The taxonomic structure of the spider community, including presence and relative abundance of individual taxa, differed between invaded and reference plots (*F*_1,26_ = 1.931; *p* = .0275) and between parks (*F*_2,26_ = 1.892; *p* = .0071).

**FIGURE 3:**
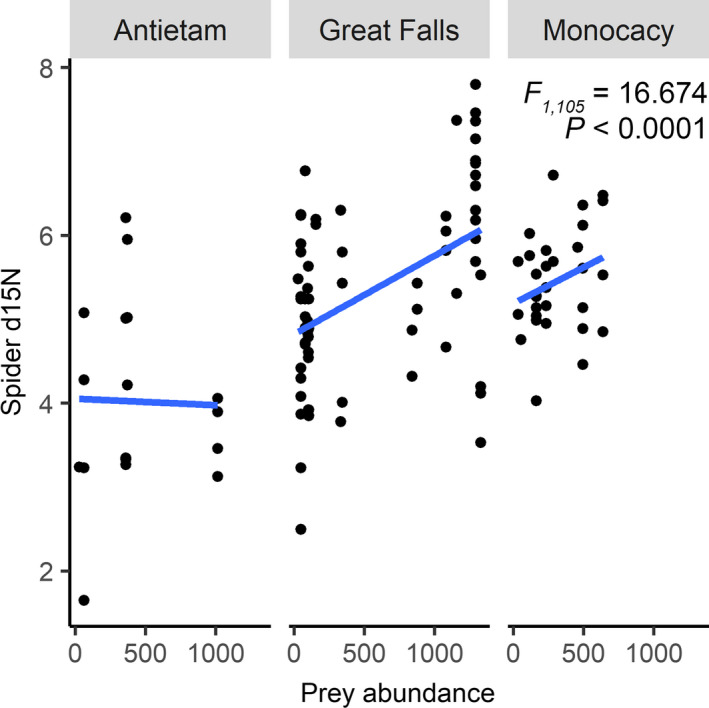
Changes in δ^15^N of sampled *Leucauge venusta* spiders across a gradient of prey density. Points represent individual sampled spiders. Positive linear relationships indicate elevated δ^15^N in plots with greater prey abundance

**FIGURE 4:**
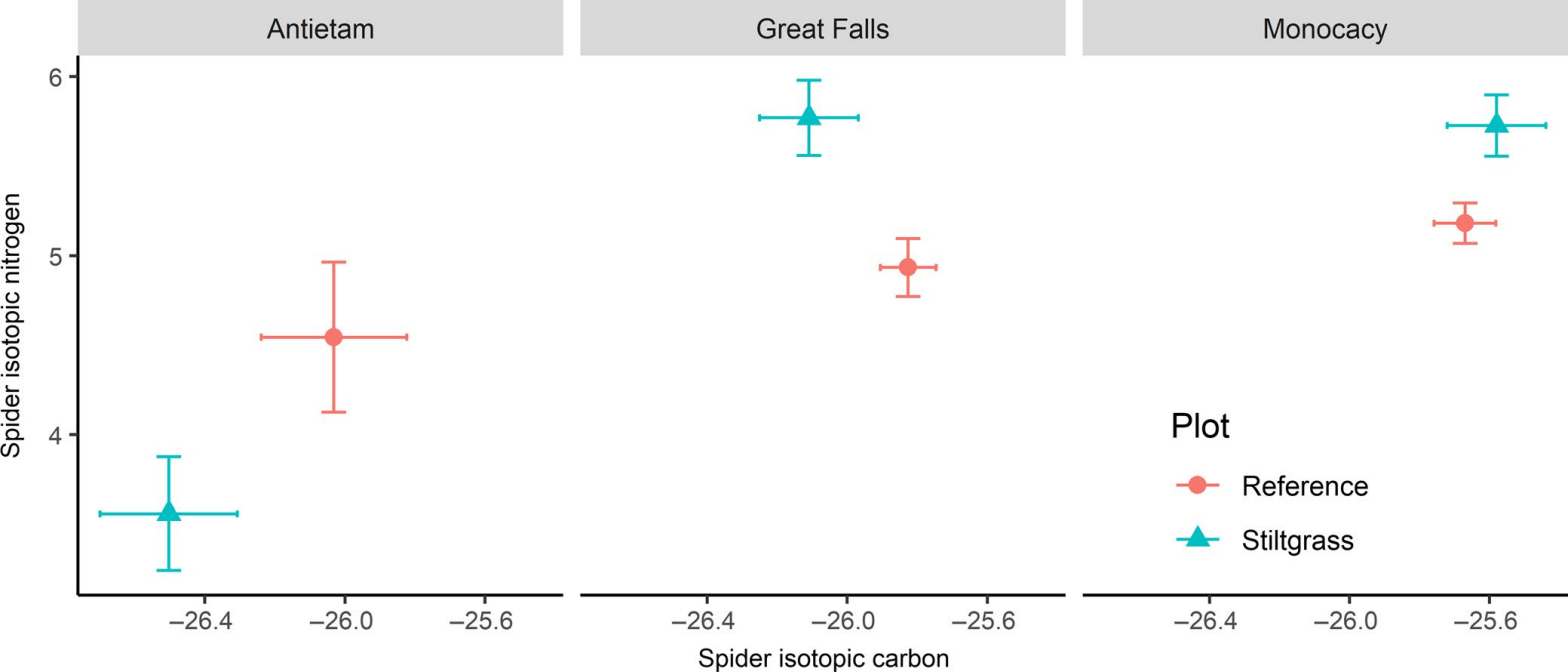
Differences in isotopic carbon and nitrogen ratios of sampled *Leucauge venusta* spiders between reference and invaded plots in the three sampled parks. Isotopic carbon results are expressed in parts per mille relative to Vienna PeeDee Belemnite, with isotopic nitrogen reported in parts per mille relative to atmospheric nitrogen. *Microstegium vimineum* typically contains δ^13^C levels between −13 and −15‰ while C_3_ plants are generally between −26 and −27‰

We collected 111 adult female *L. venusta*, with 55 individuals from *M. vimineum* plots and 56 from corresponding reference units. We sampled between 3 and 17 spiders per paired plot, with mean 3.44 ± 0.66 spiders in invaded plots and 3.50 ± 0.49 spiders from reference plots. Body mass of individual *L. venusta* differed among parks (*F*_2,105_ = 4.801; *p* = .0101), with the lowest mass at Antietam; however, individual body mass within *M. vimineum* plots was similar to that within reference plots (*F*_1,105_ = 0.288; *p* = .5930). Overall mean body mass of sampled *L. venusta* was 10.46 ± 0.67 mg per spider, with mean mass of 10.70 ± 0.73 mg in *M. vimineum* and 10.22 ± 0.61 mg in reference units. Spider mass was greater in plots with more beetles (*F*_1,105_ = 5.811; *p* = .0177) but was not related to other taxa or the presence of *M. vimineum*. Spider δ^13^C ranged from −27.78 to −24.22, with total mean −25.92 ± 0.10. Isotopic carbon values decreased with greater prey densities (*F*_1,105_ = 6.746; *p* = .0107) and flies (*F*_1,105_ = 7.689; *p* = .0066) and differed among the sampled parks (*F*_2,108_ = 6.991; *p* = .0014). Isotopic nitrogen values varied among parks (*F*_2,108_ = 12.768; *p* < .0001), with a mean value of 5.17 ± 0.18. Spider δ^15^N increased with greater prey abundance (*F*_1,105_ = 16.674; *p* < .0001; Figure 3) and exhibited similar positive correlations individually with the abundance of both beetles (*F*_1,105_ = 8.632; *p* = .0041) and flies (*F*_1,105_ = 17.264; *p* < .0001). Isotopic nitrogen values for spiders in *M. vimineum* were greater than those collected in reference plots (*F_1_*_,105_ = 4.760; *p* = .0314; Figure 4). Nitrogen values differed among parks (*F*_2,105_ = 16.624; *p* < .0001), and the interaction term between parks and plant invasion was also significant (*F*_2,105_ = 6.483; *p* = .0022), indicating that *M. vimineum* had a differing effect on isotope values across parks.

## 4 DISCUSSION

**FIGURE 5:**
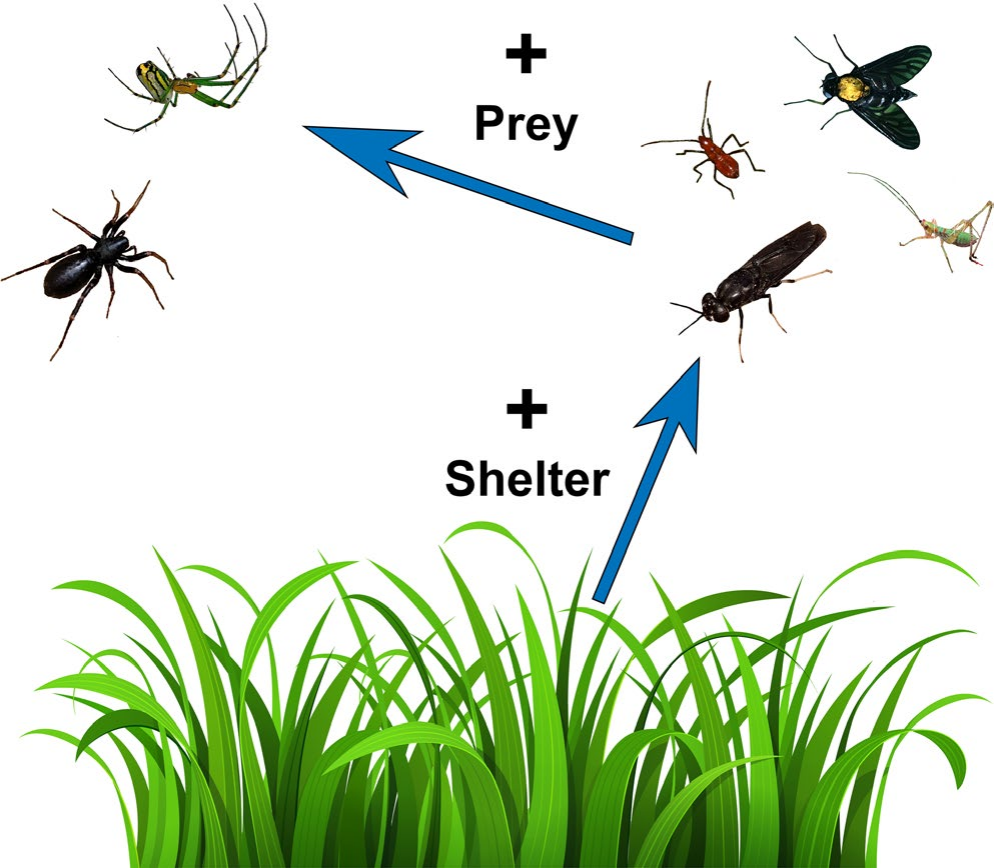
Conceptual diagram illustrating hypothesized direct and indirect effects of *Microstegium vimineum* on invertebrate groups. Presence of *M. vimineum* increases the abundance of insects by providing shelter, subsequently increasing the abundance of spiders. Photographs used with permission from R. Orr, K. Burghardt, and R. Renzi

Likely through physical changes to near‐ground structural complexity, microclimate, and leaf litter, *M. vimineum* directly and indirectly increased the abundance of nearly all understory invertebrate groups (Figure 5). The invasion of *M. vimineum* in these forested habitats directly resulted in greater local densities of insect taxa and indirectly benefited the spider community, leading to more abundant and more species‐rich spider communities. We did not detect the direct effects of understory vegetative structure on either spiders or the other components of the invertebrate community; however, our measure of structure included vegetation between 0.5 and 2.0 m from the ground. Particularly during the sampling period in our study area, *M. vimineum* provides dense, near‐ground structure below 0.5 m. Through this near‐ground structure, we found that *M. vimineum* directly benefited taxa that feed, reproduce, or develop within the detrital layer. For example, many of the dipteran species we collected belonged to taxa from detrital food webs, such as many within the Mycetophilidae. These results are similar to past research on the benefit of invasive plant species specifically to detrital communities (McCary et al., 2016) and the importance of plant structure to spider communities (Landsman & Bowman, 2017; Miyashita, Takada, & Shimazaki, 2004; Takada, Baba, Yanagi, Terada, & Miyashita, 2008).

We had also hypothesized that, due to incongruent evolutionary history, native herbivore abundance would be depressed within invaded patches. The Coleoptera were not correlated with presence of *M. vimineum*, but herbivorous Hemiptera were more abundant within the invaded plots. Given the low levels of herbivory seen on *M. vimineum* (Morrison, Lubchansky, Mauck, McCartney, & Dunn, 2007; Sanders, Belote, & Weltzin, 2004) and the general lack of understory forest vegetation in reference plots, it is likely the invaded habitats provided favorable near‐ground microhabitat conditions for invertebrates, including herbivores, without providing increased palatable plant biomass for native insects. The δ^13^C values of sampled spiders in our study area similarly reflect dietary avoidance of *M. vimineum* by herbivorous prey insects, which include the abundant planthoppers and leafhoppers found in the study area. In our study area, spiders collected in plots dominated by *M. vimineum* exhibited δ^13^C values near −26‰, while the C_4_ *M. vimineum* carbon ratios are generally between −13 and −15‰ (Bradford et al., 2010). Considering a potential stepwise increase of 0.5‰ per trophic level, it is unlikely that potential prey in this invaded habitat, including detritus‐based Diptera and phytophagous Hemiptera, are utilizing the invasive species. If so, spider δ^13^C values should be much closer to that of C_4_ plants. Given the relative abundance of flies deriving from detrital food webs in invaded plots, it is likely that the spider community in our study area is largely supported by the detritivores (Hyodo et al., 2010; McNabb, Halaj, & Wise, 2001; Miyashita, Takada, & Shimazaki, 2003). These fly larvae are likely feeding on the litter and root systems of the surrounding C_3_ trees due to the paucity of other understory plant species both within and adjacent to invaded plots. The few taxa found to utilize *M. vimineum* in the southeastern U.S. (Bradford et al., 2010) are not likely to be ingested by *L. venusta* and are too large to be captured by most forest web‐building spiders in the mid‐Atlantic U.S. The primary producers supporting the food web in this invaded habitat are the less abundant C_3_ species, indicating the importance of native vegetation in providing energy for resident herbivorous and predatory insect communities.

Our findings may be restricted to those areas with high ungulate densities and resulting depauperate understory vegetation: Deer densities in our sampling area were between 46 and 66 deer/km^2^ while densities at much lower levels can cause significant impacts to understory forest vegetation and structure (Horsley, Stout, & de Calesta, 2003; Tilghman, 1989). Vertical vegetative structure was consistently minimal in both invaded and reference plots except at Antietam, where a single reference plot contained greater vertical plant structure. This plot pair exhibited the opposite trend as compared to other plot pairs. Reference plots here contained more wasps, flies, true bugs, and, subsequently, spiders. In areas that experience a loss of plant structure from dense ungulate populations, as we found in nearly all plot pairs, *M. vimineum* may provide the only remaining shelter for insects and the only available plant structure for web‐building spiders. While we noted this in the present study with *M. vimineum*, similar trends may also arise with other invasive or native plants in areas with limited understory vegetation, given the additional structural complexity brought about by the plant. Vegetation structure is a critical factor affecting the ability of a habitat to support understory spiders in forests with extensive ungulate herbivory (Landsman & Bowman, 2017; Miyashita et al., 2004; Takada et al., 2008). In habitats where additional native or non‐native structure exists, the relationship between *M. vimineum* and the invertebrate community could reverse, as was noticeable in the Antietam plot pair and evident in the significance of the interaction term between Park and plant invasion on many of the response variables. This relationship has also been noted with forest birds in similar habitats (Tymkiw et al., 2013).

Indirectly, patches of *M. vimineum* sustain a more diverse, species‐rich, and dense spider assemblage through the subsidization of additional prey in forested habitats with substantial large vertebrate browse. Alteration of the physical environment from plant species invasion, including an enhanced structural complexity of the litter and near‐forest floor habitat, often benefits detrital consumers which in turn support forest predators (McCary et al., 2016). Forest understory spiders have been shown to benefit from augmented detrital communities (Miyashita et al., 2003), though the long‐term physiological implications from such dietary shifts are unknown. Predatory spiders are known to shift diet composition in habitats that have experienced plant species invasion and resultant changes in insect prey availability (deHart & Strand, 2012; Gratton & Denno, 2006). Flies within the invaded habitats in our study area likely contributed a greater relative proportion of the diet of understory spiders as their density within *M. vimineum* plots was nearly six times that within reference plots. Spiders also exhibited enriched δ^15^N within invaded plots and with greater abundance of Diptera, additionally indicating that detritus‐based flies, which also exhibit enriched isotopic nitrogen, constitute an increased proportion of spider diet in invaded habitats (Hyodo, 2015). As guanotelic organisms such as spiders use nitrogen efficiently, diet shifts that include a greater relative percentage of nitrogen‐poor prey may affect individual body condition and fecundity (Toft & Wise, 1999) and could cause subsequent impacts to the nutrient flow to higher predators. Spiders constitute a significant, proteinaceous component of the diet of araneophagic predators such as nesting birds. Invaded habitats may contain a greater abundance of spiders and thus more available food for forest birds; however, changes to the available spider species and nutritional quality of individual spiders may affect fecundity and development of young for birds (Ladin, D'Amico, Jaisi, & Shriver, 2015; Narango, Tallamy, & Marra, 2018; Ramsay & Houston, 2003). The patchy distribution and growth habit of *M. vimineum* populations create disparate and highly localized areas of prey subsidization for spiders, ultimately resulting in diverse and abundant predator communities. Such localized predator densities could affect forest nutritional dynamics and create hotspots of elevated nutrient levels (Hodkinson, Coulson, Harrison, & Webb, 2001; Kitchell et al., 1979; Schmitz, Hawlena, & Trussell, 2010). Changes to the arthropod food web, brought about through replacement of native plants by invasive species, are largely undocumented and potentially pervasive across forest habitats with introduced and invasive plants.

The results of this study clearly indicate the extent to which invading plant species can cause both direct and indirect effects on multiple taxa as well as different trophic and functional groups. In this particular example, the invading species benefited invertebrate communities, albeit with as yet unknown subsequent impacts on higher trophic levels. Such positive effects are certainly species‐specific and not congruent with all species invasions nor are effects consistent across affected taxa (Fletcher et al., 2019). Whether these positive effects are transient or are dependent upon current white‐tailed deer densities or the history of deer browsing requires further study. As such, management actions to control this invasive species must be conducted with consideration of deer browse and other pressures that affect understory vegetation growth, including forest succession. *M. vimineum* or other invasive species can establish on the forest floor in open canopy gaps as aging trees die, particularly in habitats such as those in our study area that contain sparse understory growth, ultimately providing additional habitat and structure for invertebrates. The importance of fully understanding the suite of effects from invasive plants, both direct and indirect and positive and negative, is apparent when considering the breadth and extent of potentially invasive plant species introductions. As the world's flora becomes increasingly homogenized from plant species introductions (Mack et al., 2000; Qian & Ricklefs, 2006), land managers and conservation biologists must consider any subsequent, cascading impacts in order to prioritize invasive plant management efforts.

## CONFLICT OF INTEREST

None declared.

## AUTHOR CONTRIBUTION

**Andrew P. Landsman:** Conceptualization (lead); Formal analysis (lead); Writing‐original draft (lead); Writing‐review & editing (equal). **Karin T. Burghardt:** Formal analysis (supporting); Writing‐review & editing (equal). **Jacob L. Bowman:** Formal analysis (supporting); Writing‐review & editing (equal).

## Data Availability

Data available from Dryad Digital Repository at https://doi.org/10.5061/dryad.7sqv9s4qs.
